# Characteristics of sawdust, wood shavings and their mixture from different pine species as bedding materials for Hanwoo cattle

**DOI:** 10.5713/ajas.19.0519

**Published:** 2019-10-22

**Authors:** Gyu Chul Ahn, Sun Sik Jang, Kang Yeon Lee, Youl Chang Baek, Young Kyoon Oh, Keun Kyu Park

**Affiliations:** 1 Department of Animal and Food Science, University of Kentucky, Lexington, KY 40506, USA; 2 National Institute of Animal Science, Rural Development Administration, Wanju 55365, Korea; 3 Semi Feed Tech. Co. Ltd., Seoul 06677, Korea; 4 Department of Animal Science and Technology, Konkuk University, Seoul 05029, Korea

**Keywords:** Animal Welfare, Bedding, Sawdust, Wood Shavings, Ammonia Emission

## Abstract

**Objective:**

This study was conducted to evaluate the physicochemical properties and changes in moisture concentrations of bedding materials under the conditions of rearing Korean Hanwoo cows.

**Methods:**

Two experiments were conducted to investigate the physicochemical characteristics (Exp. I) and usefulness as beddings for rearing cattle (Exp. II) by the type of beddings such as sawdust (SD), wood shavings (WS) and sawdust+wood shavings (S+W; 1:1 in volume), and the species of pine trees from different countries of origins (China, *Pinus armandii*, AR; Vietnam, *Pinus kesiya*, KE; USA, *Pinus rigida*, RI).

**Results:**

In Exp. I, SD-AR showed the largest proportion (78.3%) of fine particles (250 μm+ below 250 μm) and the highest bulk density (208 kg/m^3^) among treatments (p<0.05). The water absorption capacity at 24 h of both S+W-RI (713%) and -KE (701%) was the highest among treatments (p<0.05) and higher than those of SD or WS alone within each species of pine tree (p<0.05). Moisture evaporation rates (%) at 12 h were ranged from 52.3 to 60.8 for SD, 69.9 to 74.4 for WS, and 72.3 to 73.5 for S+W. Total amounts (mg/m^2^) of ammonia emissions were the lowest (p<0.05) in KE species among the pine species within each type of bedding material, having higher ability of ammonia absorption. In Exp II, KE species in both side A and B had lower moisture concentrations (%) than other species. Regardless of types of beddings except SD-AR, moisture concentrations of beddings within a pen were higher (p<0.01) at side A than B.

**Conclusion:**

The KE species has better physical characteristics than other beddings and more useful for rearing Hanwoo cattle than other beddings, probably caused by the differences in the method and degree of wood processing rather than the species.

## INTRODUCTION

Housing systems for beef cattle have a significant impact on health, welfare and performance of the animals and financially affect the farm management. Among the farm environments, beddings are crucial to maintain animal comfort in intensively managed beef cattle system indicated by the fact that provision of beddings increased essential behaviors such as lying and increased indicators of production such as weight gain [1,2].

Bedding materials are used to absorb excess moisture, improve animal comfort and re duce NH_3_ emissions, thereby alleviating negative environmental impacts from the livestock facilities. Cattle prefer to lie on soft surfaces and show longer lying time if they have access to soft surfaces [3]. The dryness of the litter is also important, and cattle are willing to lie down more on dry compared to wet bedding [3,4]. It is important, therefore, to select proper bedding materials and their management to ensure the welfare and productivity of cattle.

Different bedding materials are available in different geographic regions. Choi et al [5] surveyed housing types of Korean native cattle and beef cattle farms in Korea. The farm sized over 50 heads of cattle per farm, a total of 7,433 farms were surveyed. Most of Korean native cattle and beef cattle farms (94.7%) used litter floor rather than scraper, occupying 87.1% as sawdust barns among the litter barns. Sawdust (SD) and wood shavings (WS) itself are imported for using as a bedding material, mostly from China (*Pinus armandii*), Vietnam (*Pinus kesiya*), and USA (*Pinus rigida*).

Various physicochemical parameters such as initial mois ture content, particle size distribution, bulk density, water absorption/evaporation rate and ammonia absorption capacity are important when selecting bedding material. However, few studies have reported these characteristics of various bedding materials. Our early investigations showed that physicochemical properties of bedding materials were very different by the types (cocopeat, rice hulls, SD, and WS) and mixtures (SD and WS) of beddings [6,7].

Therefore, the comparison in the present study is of the bedding sources from different species of pine trees rather than the origin of country per se, in order that the findings are directly applicable to the beef industry. The objectives of this study were to examine the physicochemical properties of SD and WS and the mixture of SD and WS (S+W; 1:1 in volume) and to evaluate changes in moisture concentrations and the extent of moisture increment of these beddings under the condition of rearing Korean Hanwoo cows.

## MATERIALS AND METHODS

### Exp. I. The physicochemical properties of bedding materials by the species of pine trees

In Exp. I, the physicochemical properties (particle size distribution, bulk density, moisture concentration, water absorption capacity, moisture evaporation rate and *in vitro* ammonia emission) of bedding materials were evaluated by the method described by Ahn et al [6].

#### Preparation of bedding materials

Raw materials of SD and WS were pine trees imported from China (*Pinus armandii*; AR), Vietnam (*Pinus kesiya*; KE), and USA (*Pinus rigida*; RI). The S+W from each species of pine trees was mixed by 1:1 in volume, which is a typical ratio sold as a bedding material. Thus, a total of nine bedding materials (three types of bedding materials; SD, WS, and S+W ×three types of pine trees; AR, KE, and RI) were prepared for this experiment.

#### Particle size distribution, bulk density and moisture concentration

Particle size distributions of SD, WS and S+W were measured by dry sieving (Testing sieve, Chunggyesanggongsa, Seoul, Korea), mesh screen sized with 11.2, 3.35, 2.00, and 1.00 mm and 250 μm, respectively. A bedding sample of 100 g was placed in the stack of sieves, and manual shaking was performed for 10 min. After sieving, the mass retained on each sieve was weighed back. Sieving tests were replicated six times and the percentage of particle size distribution was calculated from the weight value.

Bulk density is a weight of each bedding material per vol ume unit (kg/m^3^). It was determined by adding each bedding material in a 100×100×5 cm (W×L×H) wooden box and tapping the outside of the container with a rubber mallet several times to induce light compaction by gravity. The moisture concentration of bedding materials was determined by a forced-air dry oven (SJ201D, Sejong Scientific Co., Seoul, Korea) at 105°C for overnight (over 12 h).

#### Water absorption capacity

The water absorption capacity of each bedding material was measured in a Berzelius beaker (500 mL), filtered through a filter paper (No. 417, VWR, Radnor, PA, USA) to remove remained distilled water (DW). Bedding samples for SD (30 g), WS (15 g), and S+W (25 g) were completely submerged in DW, and filtrated at 1, 2, 3, 4, 8, 12, and 24 h after submersion. At least six replications (mostly nine) were done for each bedding material. The water absorption capacity was calculated as follows;

Water absorption capacity (%)=(Wt. after submersion/Wt. before submersion)×100

#### Moisture evaporation rate 

Prior to the evaluation of moisture evaporation rates, triplicated 140 g of each bedding material were fully submerged in DW for 24 h. An electric fan (SIF-20FOG, Shinil, Seoul, Korea) was operated to evaporate moisture from bedding materials by controlling blowing distance at 2 m/s of air speed (measured by digital anemometers; AR-836, Smart Sensor, Guangdong, China), which is similar situation of typical farms in South Korea. Moisture evaporation rate was measured every hour for 12 h and calculated as a percentage rate than the amount of water absorbed and released. The moisture evaporation rate was calculated as follows;

Moisture evaporation rate (%)=(Wt. after blowing/Wt. before blowing)×100

#### In vitro ammonia emission 

Total and hourly *in vitro* ammonia emissions were measured by the laboratory chamber system and the same experimental conditions described by Ahn et al [6]. Four air-tight glass chambers (W×L×H, 26×20 ×13 cm; volume 6.76 L) for each sample were used and connected to an acid trap (100 mL of 0.9 M sulfuric acid; [8]) to collect ammonia emission from the mixture of beddings (400 g), feces (200 g) and water with 60% of moisture concentrations. The amount of feces added to beddings was equivalent to 1% of the daily excretion by Hanwoo cows. Daily excretion of a cow (487 kg) were approximately 20 kg, including 15.5 kg of feces and 4.6 L of urine, respectively [9].

An air pump (13 watts, YP-15A, Youngnam Air Pump Inc., Busan, Korea) was used to send ammonia emission gases from the beddings in the chamber to the outlet acid trap. Inlet acid traps were also installed to remove ammonia gas from the outside air and to prevent beddings from becoming too dry. The air flow rate into each chamber was adjusted to 4 L/min using gas flow meters (RMA-26-SSV, Dwyer, Leesburg, VA, USA), thus the turnover rate of each chamber was approximately once per minute. The height of the mixture of beddings, feces and water in a measuring chamber was approximately 5 cm, which is a typical bedding height at farms in Korea. This experiment was conducted at a constant room temperature (25°C).

Total and hourly ammonia emission rates were measured at 1, 3, 6, 12, 24, and 36 h after operating the air pump by replacing fresh acid traps at each sampling time. Ammonia emission (*F*, mg of N/m^2^/h) were calculated as;

F=XV/At

Where, *X* is ammonia-N (NH_3_-N) concentration in an acid trap (mg/L), *V* is the volume of acid trap solution (L), *A* is the exposed surface area of a chamber (m^2^) and *t* is the duration of sampling period (h).

### Exp. II. The evaluation of bedding materials by the species of pine trees for rearing Hanwoo cattle

#### Experimental design

All procedures employed during the research were approved by the Institutional Animal Care and Use Committee at Konkuk University (Approval number: KU16108). In Exp. II, a total of nine bedding materials (three types of bedding materials; SD, WS, and S+W ×three types of pine trees; AR, KE, and RI) were evaluated to estimate the usability of bedding materials for Hanwoo cow. Seventy-two Hanwoo cows (492±38 kg) with more than third-parity in the dry (non-lactating) period were allocated to each of nine types of bedding materials and an experimental period was 21 days, and performed duplicated in 42 days (6 weeks, October to November). Eighteen pens were allocated in nine bedding materials, duplicated for each treatment. After 3-week of experimental periods, all beddings were replaced with fresh ones for duplicated results. The animals were housed in a pen (4 cows/pen; 4.0 m wide×8.2 m length = 32.8 m^2^/pen; 8.2 m^2^ for each cow) with one side on 4.0 m wide feed bunk side (side A) and another side equipped with water supply (side B). There was a shallow concrete divider (15 cm height×20 cm wide) between side A and B within each pen.

Offered feeds were in the form of total mixed ration and the chemical composition of the diet is presented in Table 1. Diets were offered 10 kg (as fed basis) on a daily basis at 06:00 and 17:00 h to prevent overweight that could affect reproduction efficiency of cows. The cows were allowed to access fresh water and mineral block without any restriction during the whole experimental period. Blowing fans (diameter = 1,025 mm, 790 rpm; DVN-1007, Dongkun Industrial Co., Ltd, Incheon, Korea) were installed every two pens with wind speed at 2 m/s and working 24 h for maximizing the usability of bedding materials. Prior to the bedding trial, the existing litter was removed and installed fresh beddings according to each treatment at a height of 5 cm. Kang et al [10] suggested that 5 cm thickness bedding was the most economical among 5, 10, 15, and 20 cm thickness of SD.

#### Sampling and analysis of beddings

Beddings samples were collected every week at 10:00 AM from 12 sampling spots per pen by grab sampling to avoid sampling bias. Obtained samples were transported immediately to the laboratory for measuring moisture concentrations using a forced-air dry oven at 105°C for more than 12 h.

#### Statistical analysis

Data obtained from the evaluation of physicochemical properties and changes in moisture concentration of bedding materials under the condition of rearing for Hanwoo cattle were subjected to statistical analysis using the general linear model procedure of SAS (SAS Inst. Inc., Cary, NC, USA). Data were analyzed by analysis of variance and Duncan’s multiple range tests to determine significant differences (p<0.01 and 0.05) among treatments.

## RESULTS AND DISCUSSION

### Exp. I. The physicochemical properties of bedding materials by the species of pine trees

#### Particle size distribution, bulk density and moisture concentration

The particle size distribution, bulk density and moisture concentration of bedding materials by the species of pine trees are shown in Table 2. In sieve size with 11.2 mm, which was the largest pore size among sieves, WS-RI showed the highest percentage (12.8%) among treatments (p<0.05). Other bedding materials except S+W-RI (3.1%) showed almost 0% of particles with 11.2 mm. The WS-AR and -KE treatments showed higher proportions than other treatments on sieve size with 3.35 mm (53.2% and 51.0%, respectively; p<0.05) and 2.00 mm (21.3% and 22.3%, respectively; p<0.05). On the contrary, the SD-KE (36.5%) and -RI (36.9%) had the highest percentage on particle size with 1.00 mm among treatments (p<0.05). Overall, SD had lower proportions of less than 1mm particles compared to WS treatments. Intermediate values were observed for S+W within each species of pine trees, but the values were not exactly half of SD and WD because the mixing ratio was based on the volume (1:1) rather than the weight of bedding materials. In practical, mixing of different types of bedding materials by volume may be more applicable than the weighing of bedding material prior to use in a farm.

In particle size of 250 μm and below 250 μm, SD-AR showed the largest proportion (78.3%), followed by S+W-AR (55.8%) among treatments (p<0.05). The values from other SD treatments, i.e. for SD-KE and -RI were 47.3% and 45.5%, respectively. Ahn et al [6] suggested that more than 60% of small particles (250 μm + <250 μm) in a bedding material could generate a dust problem in on-farm trial. Other researchers reported similar results. For example, particles less than 300 μm tends to make a dust problem in rodent beddings [11]. Equine stalls bedded with flax shive (79% of less than 2 mm particles of total mass) were relatively dustier than those with pine WS (65% of more than 6.3 mm particle) [12]. Moreover, particle size distribution is a significant factor influencing the bulk density and water absorption capacity [13]. Therefore, SD-AR and S+W-AR may not be suitable for bedding materials.

Bulk densities were the highest in SD-AR (208 kg/m ^3^), followed by S+W-AR (184 kg/m^3^) among treatments (p<0.05). Generally, bulk density increases as particle size decreases because the volume of spaces between particles decreases. Thus, the high bulk density of SD-AR and S+W-AR was results from the high proportion of particles with 250 μm + <250 μm. As expected, bulk densities of WS was lower (p<0.05) than those of SD and S+W regardless of the species of pine trees. Low bulk density generally has the disadvantage of increasing the transport cost and reducing the capacity for storage. Conversely, very low values cause a decline in water absorption capacity of the beddings and, concomitantly leading to more wet floor. The bulk density ratio between WS and SD was 63%, 74%, and 77% for AR, KE and RI, respectively, whereas the ratio between S+W and SD increased to 88%, 99%, and 96% for the species, respectively. Thus, mixing of two types of beddings may help to improve bulk density.

Initial moisture concentration of bedding materials was the highest in SD-RI (17.9%; p<0.05) and the lowest in WS-AR among treatments (9.7%; p<0.05). However, the numerical difference was not large, the values may vary depending on weather, processing method and storage condition of the beddings.

#### Water absorption capacity

The water absorption capacity of bedding materials by the species of pine trees is shown in Table 3. The WS-AR (237%) and -KE (291%) treatments after immersing in water for 1 h showed the lowest water absorption capacity among treatments (p<0.05), whereas SD-KE (604%) and WS-RI (551%) were the highest (p<0.05). Water holding capacity of a material is defined as the amount of water that a material can hold at the point of saturation. The water holding capacity of any material varies mainly due to differences in the degree of grinding, altering the particle size distribution and surface area. Organic matter with fine particles absorbed significantly more water than coarse and medium ground particles of the same bedding material [13]. Therefore, coarsely ground particles such as WS had a lower water holding capacity than SD, particularly during the initial time of submersion (1 to 4 h), because it had less surface area to absorb and hold water. However, this fact may be applicable at the initial absorption stage because changes in the absorption rate with advancing times, especially after 8 h, were varied depending on types of beddings (SD vs WS) and the pine species, probably due to varying degrees of particle size distribution and bulk densities.

After 24 h of soaking in the water, the water absorption capacity of bedding materials was ranged from 511% (SD-AR) to 713% (S+W-RI). Both S+W-RI (713%) and -KE (701%), except S+W-AR (543%) which had high proportion of extremely small particles, were the highest among treatments (p<0.05), and higher than those of SD or WS alone within each species of pine trees (p<0.05). No statistical difference was detected between S+W-RI and -KE. Therefore, mixing of SD and WS was more advantageous than the single use.

Overall, the observed values were higher than those of our earlier investigations [6,7]. In those two studies, water absorption capacities at 24 h were 444% for SD [6], 270% SD, and 540% WS, respectively [7]. Both SD and WS in earlier studies were obtained from a commercial wood-processing company in Korea, originated from Russia (*Pinus sylvestris*) and New Zealand (*Pinus radiata*). In this study, the beddings were imported as SD or WS from other countries, solely for the purpose of bedding materials. The discrepancy could be caused by the differences in raw material of bedding sources, i.e., pine trees and the extent of wood processing. In this study, relatively large variations in particle size distribution, bulk density and water absorption capacity of bedding materials by the species were observed. One of the important considerations in beddings is the rate and capacity of water absorption to absorb urine and ammonia [14]. It is also important for keeping the animals clean and comfortable, thereby improving animal welfare [1].

#### Moisture evaporation rate

The moisture evaporation rate (%) of bedding materials by the species of pine trees is shown in Table 4. After blowing air at 2 m/s after 1 to 4 h, WS had higher moisture evaporation rates (p<0.05) than those of SD and S+W, probably due to the higher porosity of WS. At 5 h, WS was not statistically different from SD and S+W except for SD-AR.

At 6 h, only RI among the species of pines was not statisti cally different by the types of beddings (SD, WS, and S+W). However, the other WS and S+W treatments from 6 to 12 h showed higher evaporation rates (p<0.05) than SD regardless of the species of pine trees (AR, KE, and RI). No differences were detected between the WS and S+W treatments among treatments after this time. Interestingly, the SD-RI showed higher moisture evaporation rate (p<0.05) than those of other SD treatments after 6 h. This might be associated with the high proportion of large particles of 3.35 mm which is the particle size occupying the highest proportion in WS. The proportion on 3.35 mm-sieve of SD-RI (8.0%) were higher (p<0.05) than those of SD-AR (0.1%) and SD-KE (0.8%). Conversely, the proportion on 2.00 mm of SD-RI (9.7%) was lower (p<0.05) than that of SD-KE (15.5%) but higher (p<0.05) than that of SD-AR (1.5%).

Moisture evaporation rates at 12 h were ranged from 52.3% to 60.8% for SD, 69.9% to 74.4% for WS, and 72.3% to 73.5% for S+W. Under the same procedure of experiment, our earlier studies reported different results, in which the moisture evaporation rate at 12 h was 71.2% for SD [6], 70.5% SD, 75.4% WS, and 72.2% S+W, respectively [7]. These results suggest that physical characteristics of bedding materials are mostly affected by the type or shape of beddings rather than the species of pine trees. The characteristics of the bedding materials should not only be important the ability to absorb moisture quickly, that is the water absorption capacity, but also the ability to rapidly evaporate the moisture it contains. Taking this into consideration, simply mixing of SD and WS (i.e., S+W) could lead to more favorable changes in bedding properties rather than using one type of bedding. Conventionally, the mixing ratio of SD and WS depends on the availability and the extent of dust problem of these materials in a bedding company. In most farms, it has been used even without discriminating between SD and WS.

#### In vitro ammonia emission

*In vitro* ammonia emission rates (mg/m^2^/h) and total ammonia emissions (mg/m^2^) of bedding materials by the species of pine trees are shown in Table 5 and 6, respectively. Ahn et al [6] suggested that estimating ammonia emissions at actual farm level may be incorrect because of weather conditions and other factors such as wind, humidity, temperature and amount of fecal load. After 1 h of operating *in vitro* ammonia emission chamber, WS showed the highest ammonia emission rates (p<0.05) but did not show statistical differences with SD-AR and -RI, and S+W-KE. On the other hand, after operating *in vitro* chamber for 3 h, both WS-KE and -RI showed lower ammonia emissions (p<0.05) than other treatments except WS-AR and S+W-RI.

The ammonia emissions were declined dramatically from 1 to 3 h for WS (6.11 to 2.43, WS-AR; 5.10 to 1.24, WS-KE; 5.61 to 1.73, WS-RI), then decreased closed to zero. For other bedding materials, decreases in emission were observed at 6 h and reached almost zero after 12 h. Within 6 to 12 h of emission times, WS showed lower rates of ammonia emission (p< 0.05) than others. After 24 h, *in vitro* ammonia emission rates were near to zero values and did not show statistical differences among the whole treatment. Average ammonia emission rates for 36 h were lower (p<0.05) in WS-KE (1.07 mg/m^2^/h), RI (1.35), and S+W-KE (0.97) than the others, but not statistically different with SD-KE (1.44), WS-AR (1.44), and S+ W-RI (1.49). Intermediate values were observed for S+W between SD and WS within each species of pine trees.

Total ammonia emission (mg/m ^2^), meaning the total amount of ammonia emission from initial to each sampling time, seems to be a better index to compare the ammonia absorption capacity of beddings. Final ammonia emissions at 36 h were the lowest (p<0.05) in WS-KE (7.79 mg/m^2^) and S+W-KE (7.87), followed by the order of WS-AR (11.24) = WS-RI (11.56) < SD-KE (16.87) = S+W-RI (19.45) < SD-RI (30.64) < SD-AR (38.84) = S+W-AR (40.79). The reason for high values of SD-AR and S+W-AR is probably due to the high proportion of fine particles with 250 μm + <250 μm.

The KE showed the lowest total ammonia emissions (p < 0.05) among the pine species within each type of bedding material, having higher ability of ammonia absorption. In addition, WS showed lower amount of total ammonia emission (p<0.05) than other treatments as time progressed, probably due to higher proportions of large particles and thereby lowering the surface area to volume ratio to emit ammonia. According to Misselbrook and Powell [14], the physical characteristics such as urine absorbance capacity and bulk density were more important than their chemical properties (pH, cation exchange capacity, and C:N) of bedding materials, because ammonia emissions increased linearly with absorbance capacity and decreased as the bulk density of the packed beddings increased.

Based on the results from this study, S+W has better physi cal characteristics than SD or WS alone, considering higher water absorption capacity than SD and similar bulk density, moisture evaporation rate and ammonia absorption capacity with WS. Using SD alone is not recommended because of high proportions of fine particle and other inferior physicochemical properties. Overall, KE has better characteristics than other species of pine trees, having proper level of water absorption/evaporation rate and high capacity of ammonia absorption.

### Exp. II. The evaluation of bedding materials by the species of pine trees for rearing Hanwoo cattle

#### Moisture concentration

The moisture concentrations (%) of bedding materials by the species of pine trees are shown in Table 7. There were no significant 2-way and 3-way interactions among experimental period, bedding type and pen location (feed bunk side A and waterer side B within a pen) during the whole experimental period. The WS-AR showed the lowest moisture concentration (p<0.05) among treatments at side A throughout the entire period, and also showed the lowest concentrations (p<0.05) at side B during 2 to 3 weeks. As a result, average moisture concentrations of WS-AR in both side A and B were lower (p<0.05) than any other treatments, reflecting relatively high rate of moisture evaporation.

In side A at the first week of experiment, moisture concen trations of KE species of pines, regardless of type of beddings, were lower (p<0.05) than RI. When comparing AR species, WS-AR (28.7%) was lower but S+W-AR (52.1%) was higher than KE (p<0.05), and SD-AR (43.6%) had no statistically significant difference from KE. At week 2, the lowest value was observed in WS-AR (52.3%) and the highest value was in S+W-RI (65.7%) which was already surpassed 65% moisture within two weeks. The rest of other treatments were not statistically different. At week 3, moisture concentrations of all treatments except WS-AR (49.6%) and SD-KE (63.4%) reached more than 65%, meaning bedding replacement is required [15].

In side B at week 1, moisture concentrations of WS except SD-KE and -RI were lower (p<0.05) than the rest of other treatments. At week 2, WS treatments were also lower (p<0.05) than SD and S+W. At week 3, moisture concentrations of SD-KE, S+W-KE, and all WS treatments were less than 65% but those of other treatments (SD-AR, -SI, and S+W) exceeded 65%. Particularly in places with low urinal and fecal loads, such as side B, WS with large particles having high moisture evaporation rate may help to keep the moisture content low.

Average moisture concentration of WS-AR (43.5%) during the whole experimental period was the lowest (p<0.05) but variable for other treatments by the type and species with no distinct tendency at side A. However, at side B, SD-AR (55.6%), S+W-AR (53.3%), and -RI (57.0%) showed higher average moisture concentrations (p<0.05) than other treatments. Overall, KE species in both side A and B had lower moisture concentrations than other species, reflecting better physicochemical properties.

When comparing side A and B, average moisture concen trations of side A within a pen were higher (p<0.01) than those of side B among treatment except SD-AR because cows spend most of their time at side A for feeding. These results are consistent with our previous studies [6,7]. Therefore, for proper bedding management, the manure load of side A should be reduced to the extent of side B by increasing replacement cycle of beddings or bedding thickness (more than 5 cm). However, the determination of an appropriate thickness that does not interfere with the rate of water evaporation remains as a further study.

#### Moisture increment

The moisture increment (% unit) of bedding materials by the species of pine trees are presented in Table 8. Moisture increment was calculated by subtracting the moisture concentration at each week from the value of the week before. As shown in the moisture content results, WS-AR (side A, 13.3; B, 12.4) of average moisture increment were lower (p<0.05) than other treatments; the treatment showed negative moisture increment (−2.8, side A) at the third week.

At side A, average moisture increments of AR, KE, and RI were not statistically different in SD, WS and S+W, except lower (p<0.05) in WS-AR than the rest of other treatments. At side B, WS showed lower average moisture increment (p< 0.05) than other treatments except SD-KE, having intermediate value. Overall, the moisture increment for each experimental week did not show a clear tendency and was variable by the type and species of beddings, probably due to various physicochemical properties.

Average moisture increments of KE species at side A were higher (p<0.01) than that of side B. However, no statistically significant difference was observed for other species. Summarizing the results of moisture concentrations and increment by the types of bedding materials trial, KE showed better performances than other species. However, this seems to be due to the physicochemical differences in the method and degree of wood processing, rather than the differences in species.

## CONCLUSION

Bedding materials have variable physicochemical characteristics by the type and species of bedding materials. Therefore, the ideal selection of proper bedding materials should be based on its physicochemical properties, not solely by the species, the price or availability. Using the mixture of SD and WS (1:1 in volume) would be a better option for effective utilization of bedding materials, considering lower proportion of fine particles than SD and higher bulk density than WS with relatively good moisture evaporation rate.

## Figures and Tables

**Table 1:** Chemical composition of experimental diet

Items	Concentration (%)
Dry matter	59.90
	--------------- % of DM -------------
Crude protein	15.28
Ether extract	3.34
Crude fiber	17.89
Ash	8.60
Neutral detergent fiber	39.76
Acid detergent fiber	20.50
Lignin	2.86
Ca	0.84
P	0.44

**Table 2:** Particle size distribution, bulk density and moisture concentration of bedding materials according to the species of pine trees

Item	Particle size distribution1) (%)									SEM

	SD			WS			S+W			
		
	AR	KE	RI	AR	KE	RI	AR	KE	RI	
Particle size										
11.2 mm	0.00^c^	0.00^c^	0.00^c^	0.15^c^	0.20^c^	12.75^a^	0.00^c^	0.00^c^	3.10^b^	0.26
3.35 mm	0.10^f^	0.75^f^	7.95^e^	53.15^a^	51.00^a^	46.35^b^	15.25^d^	17.10^d^	22.75^c^	0.07
2.00 mm	1.50^f^	15.45^c^	9.70^de^	21.25^a^	22.25^a^	10.85^d^	10.10^de^	18.55^b^	8.95^e^	0.14
1.00 mm	20.10^d^	36.50^a^	36.85^a^	17.55^f^	15.55^g^	14.05^h^	18.90^e^	27.25^b^	25.75^c^	0.01
250 μm	63.10^a^	35.35^d^	38.40^c^	2.55^i^	5.50^h^	11.05^g^	43.00^b^	26.00^f^	29.25^e^	0.12
<250 μm	15.20^a^	11.95^ab^	7.10^cd^	5.35^d^	5.50^d^	4.95^d^	12.75^ab^	11.10^b^	10.20^bc^	0.01
250 μm+<250 μm	78.30^a^	47.30^c^	45.50^c^	7.90^f^	11.00^f^	16.00^e^	55.75^b^	37.10^d^	39.45^d^	0.01
Bulk density (kg/m^3^)	208^a^	173^c^	151^d^	132^e^	128^e^	117^f^	184^b^	171^c^	145^d^	0.81
Moisture (%)	13.87^b^	11.69^c^	17.85^a^	9.70^d^	10.63^c^	11.28^c^	12.52^bc^	10.38^c^	14.54^b^	0.30

SEM, standard error of means.

1) SD, sawdust; WS, wood shavings; S+W, mixture of sawdust and wood shavings (1:1 in volume); AR, *Pinus armandii* (from the China); KE, *Pinus kesiya* (from the Vietnam); RI, *Pinus rigida* (from the USA).

a–i Means within a row without a common superscript letter differ (p<0.05).

**Table 3:** The water absorption capacity of bedding materials according to the species of pine trees

Time (h)	Water absorption capacity1) (%)									SEM

	SD			WS			S+W			
		
	AR	KE	RI	AR	KE	RI	AR	KE	RI	
1	483.6^bc^	683.5^a^	487.8^bc^	236.5^e^	291.3^de^	550.8^ab^	500.9^bc^	478.9^bc^	394.3^cd^	9.29
2	511.6^cd^	609.1^a^	563.3abc	399.4^e^	439.6^e^	542.4bcd	503.6^d^	571.4^ab^	549.0bcd	8.48
4	492.2^cd^	611.1^ab^	555.7abc	385.0^e^	438.8^de^	531.5^c^	514.9^c^	621.3^a^	545.4^bc^	9.49
8	508.9^de^	612.5^ab^	545.4bcd	451.5^e^	493.3^de^	587.8abc	535.3^cd^	629.5^a^	633.6^a^	7.73
12	508.9^c^	617.6^b^	580.4^bc^	523.8^c^	564.7^bc^	604.0^b^	545.2^bc^	694.8^a^	712.6^a^	9.18
24	511.0^d^	619.4^c^	577.2^cd^	556.3^cd^	565.1^cd^	614.5^bc^	543.4^cd^	701.0^ab^	712.8^a^	9.97

SEM, standard error of means.

1) SD, sawdust; WS, wood shavings; S+W, mixture of sawdust and wood shavings (1:1 in volume); AR, *Pinus armandii* (from the China); KE, *Pinus kesiya* (from the Vietnam); RI, *Pinus rigida* (from the USA).

a–e Means within a row without a common superscript letter differ (p<0.05).

**Table 4:** The moisture evaporation rate of bedding materials by the species of pine trees from water-saturation by blowing air at 2 m/s

Time (h)	Water evaporation rate1) (%)									SEM

	SD			WS			S+W			
		
	AR	KE	RI	AR	KE	RI	AR	KE	RI	
1	7.71^c^	6.48^c^	7.50^c^	13.97^a^	12.54^a^	10.38^b^	7.09^c^	7.12^c^	7.36^c^	1.03
2	13.45^c^	11.42^c^	12.77^c^	21.22^a^	19.60^a^	16.84^b^	11.83^c^	11.88^c^	12.28^c^	1.02
3	27.66^c^	24.96^d^	26.78^cd^	34.22^a^	33.61^a^	31.14^b^	25.64^cd^	25.64^cd^	26.20^cd^	0.71
4	31.20^b^	28.55^c^	30.35^bc^	33.29^a^	34.88^a^	33.77^a^	29.16^bc^	29.16^bc^	29.26^bc^	0.63
5	38.97^b^	42.91^a^	43.95^a^	43.47^a^	41.11^ab^	42.76^a^	41.71^ab^	41.82^ab^	42.40^a^	0.55
6	34.34^c^	38.36^b^	42.05^a^	44.62^a^	42.99^a^	44.26^a^	43.46^a^	43.58^a^	44.01^a^	0.62
7	37.58^c^	40.67^c^	47.03^b^	54.32^a^	54.73^a^	54.80^a^	53.65^a^	54.87^a^	54.77^a^	0.73
8	40.41^c^	43.73^c^	49.29^b^	54.82^a^	54.16^a^	54.87^a^	54.38^a^	54.54^a^	54.73^a^	0.71
9	43.39^c^	45.58^c^	52.66^b^	61.59^a^	64.79^a^	63.07^a^	63.83^a^	64.25^a^	63.41^a^	0.81
10	44.96^c^	47.80^c^	54.54^b^	63.02^a^	64.94^a^	64.10^a^	64.42^a^	64.73^a^	64.23^a^	1.02
11	51.28^d^	52.25^d^	58.32^c^	67.89^b^	73.77^a^	70.12^ab^	71.94^ab^	72.42^ab^	70.82^ab^	1.02
12	52.26^c^	54.31^c^	60.83^b^	69.94^a^	74.36^a^	71.77^a^	72.94^a^	73.49^a^	72.25^a^	1.11

SEM, standard error of means.

1) SD, sawdust; WS, wood shavings; S+W, mixture of sawdust and wood shavings (1:1 in volume); AR, *Pinus armandii* (from the China); KE, *Pinus kesiya* (from the Vietnam); RI, *Pinus rigida* (from the USA).

a–d Means within a row without a common superscript letter differ (p<0.05).

**Table 5:** Ammonia emission of bedding materials by the species of pine trees measured by the chamber system

Time (h)	Ammonia emission rate1) (mg/m^2^/h)									SEM

	SD			WS			S+W			
		
	AR	KE	RI	AR	KE	RI	AR	KE	RI	
1	4.27^ab^	3.17^b^	4.07^ab^	6.11^a^	5.10^a^	5.61^a^	3.22^b^	4.30^ab^	3.52^b^	1.01
3	4.10^a^	3.70^a^	4.00^a^	2.43^ab^	1.24^b^	1.73^b^	3.92^a^	1.11^b^	2.82^ab^	1.02
6	1.99^a^	1.10^a^	1.79^a^	0.09^b^	0.07^b^	0.73^ab^	2.11^a^	0.37^ab^	1.71^a^	0.71
12	1.20^a^	0.50^b^	0.80^ab^	0.00^c^	0.00^c^	0.05^c^	1.26^a^	0.04^c^	0.86^ab^	0.13
24	0.50	0.00	0.30	0.00	0.00	0.00	0.61	0.00	0.00	0.08
36	0.60	0.00	0.40	0.00	0.00	0.00	0.71	0.00	0.00	0.09
Mean	2.11^a^	1.44^ab^	1.89^a^	1.44^ab^	1.07^b^	1.35^b^	1.89^a^	0.97^b^	1.49^ab^	0.51

SEM, standard error of means.

1) SD, sawdust; WS, wood shavings; S+W, mixture of sawdust and wood shavings (1:1 in volume); AR, *Pinus armandii* (from the China); KE, *Pinus kesiya* (from the Vietnam); RI, *Pinus rigida* (from the USA).

a–c Means within a row without a common superscript letter differ (p<0.05).

**Table 6:** Total ammonia emission of bedding materials by the species of pine trees measured by the chamber system

Time (h)	Total ammonia emission rate1) (mg/m^2^)									SEM

	SD			WS			S+W			
		
	AR	KE	RI	AR	KE	RI	AR	KE	RI	
1	4.27^ab^	3.17^b^	4.07^ab^	6.11^a^	5.10^a^	5.61^a^	3.22^b^	4.30^ab^	3.52^b^	1.01
3	12.47^a^	10.57^a^	12.07^a^	10.97^a^	7.58^b^	9.07^ab^	11.06^a^	6.52^b^	9.16^ab^	1.47
6	18.44^a^	13.87^b^	17.44^a^	11.24^b^	7.79^c^	11.26^b^	17.39^a^	7.63^c^	14.29^ab^	1.87
12	25.64^a^	16.87^b^	22.24^ab^	11.24^c^	7.79^d^	11.56^c^	24.95^a^	7.87^d^	19.45^b^	1.14
24	31.64^a^	16.87^c^	25.84^b^	11.24^d^	7.79^e^	11.56^d^	32.27^a^	7.87^e^	19.45^c^	1.27
36	38.84^a^	16.87^c^	30.64^b^	11.24^d^	7.79^e^	11.56^d^	40.79^a^	7.87^e^	19.45^c^	1.57

SEM, standard error of means.

1) SD, sawdust; WS, wood shavings; S+W, mixture of sawdust and wood shavings (1:1 in volume); AR, *Pinus armandii* (from the China); KE, *Pinus kesiya* (from the Vietnam); RI, *Pinus rigida* (from the USA).

a–e Means within a row without a common superscript letter differ (p<0.05).

**Table 7:** Effects of bedding materials by the species of pine trees and pen location on moisture concentrations of beddings for rearing Hanwoo

Side	Week	Moisture concentration1) (%)									SEM

		SD			WS			S+W			
		
		AR	KE	RI	AR	KE	RI	AR	KE	RI	
A, B2)	0	13.9^b^	11.7^c^	17.6^a^	9.7^d^	10.6^c^	11.3^c^	12.5^bc^	10.4^c^	14.5^b^	0.30
A	1	43.6^ab^	36.6^b^	49.2^a^	28.7^c^	39.7^b^	46.4^a^	52.1^a^	37.6^b^	46.8^a^	3.02
	2	55.9^b^	58.7^b^	56.8^b^	52.3^c^	59.7^b^	60.4^b^	59.9^b^	61.6^b^	65.7^a^	2.04
	3	66.6^b^	63.4^b^	75.9^a^	49.6^c^	72.7^a^	68.2^b^	75.7^a^	72.7^a^	74.3^a^	2.34
	Mean	55.7^b^	52.9^b^*	60.6^a^*	43.5^c^*	57.4^ab^*	58.3^a^*	62.5^a^*	57.3^ab^*	62.3^a^*	2.47
B	1	40.7^a^	22.3^c^	28.6^b^	26.9^b^	30.0^b^	29.1^b^	37.3^a^	39.1^a^	40.4^a^	2.30
	2	55.8^a^	47.2^b^	47.8^b^	30.7^d^	40.1^c^	43.1^c^	54.6^a^	49.7^b^	59.4^a^	2.41
	3	70.3^a^	57.8^b^	71.2^a^	46.9^c^	51.0^c^	52.1^c^	67.9^a^	62.4^b^	71.1^a^	3.68
	Mean	55.6^a^	42.4^b^*	49.2^b^*	34.9^d^*	40.4^c^*	41.4^c^*	53.3^a^*	50.4^ab^*	57.0^a^*	2.80

SEM, standard error of means.

1) SD, sawdust; WS, wood shavings; S+W, mixture of sawdust and wood shavings (1:1 in volume); AR, *Pinus armandii* (from the China); KE, *Pinus kesiya* (from the Vietnam); RI, *Pinus rigida* (from the USA).

2) A, feed bunk side within a pen; B, waterer side within a pen.

a–d Means within a row without a common superscript letter differ (p<0.05).

* Means between side A and B differ (p<0.01).

**Table 8:** Effects of bedding materials by the species of pine trees and pen location on moisture increment of beddings for rearing Hanwoo

Side	Week	Moisture increment1) (% unit)									SEM

		SD			WS			S+W			
		
		AR	KE	RI	AR	KE	RI	AR	KE	RI	
A, B2)	0	13.9^b^	11.7^c^	17.9^a^	9.7^d^	10.6^c^	11.3^c^	12.5^bc^	10.4^c^	14.5^b^	0.30
A	1	29.7^b^	24.9^bc^	31.4^b^	19.0^c^	29.1^b^	35.1^ab^	39.6^a^	27.2^bc^	32.2^b^	1.21
	2	13.4^b^	22.2^a^	7.6^c^	23.6^a^	20.0^ab^	14.1^b^	7.8^c^	24.0^a^	18.9^ab^	1.17
	3	9.7^c^	4.6^d^	19.1^a^	−2.8^e^	13.0^b^	7.8^c^	15.8^b^	11.1^bc^	8.6^c^	1.19
	Mean	17.6^a^	17.2^a^*	19.3^a^	13.3^b^	20.7^a^*	19.0^a^*	21.0^a^	20.8^a^*	19.9^a^	1.19
B	1	26.8^a^	10.6^c^	10.7^c^	17.2^b^	19.3^b^	17.8^b^	24.8^a^	28.7^a^	25.9^a^	1.47
	2	15.1^ab^	24.9^a^	19.3^a^	3.8^c^	10.1^b^	14.0^ab^	17.3^a^	10.6^b^	19.0^a^	1.12
	3	14.5^b^	10.6^c^	23.4^a^	16.3^b^	10.9^c^	9.0^c^	13.2^bc^	12.7^bc^	11.7^c^	1.27
	Mean	18.8^a^	15.4^ab^*	17.8^a^	12.4^b^	13.4^b^*	13.6^b^*	18.4^a^	17.4^a^*	18.9^a^	1.29

SEM, standard error of means.

1) SD, sawdust; WS, wood shavings; S+W, mixture of sawdust and wood shavings (1:1 in volume); AR, *Pinus armandii* (from the China); KE, *Pinus kesiya* (from the Vietnam); RI, *Pinus rigida* (from the USA).

2) A, feed bunk side within a pen; B, waterer side within a pen

a–e Means within a row without a common superscript letter differ (p<0.05).

* Means between side A and B differ (p<0.01).
